# Testing the utility of GPT for title and abstract screening in environmental systematic evidence synthesis

**DOI:** 10.1186/s13750-025-00360-x

**Published:** 2025-04-23

**Authors:** Björn Nykvist, Biljana Macura, Maria Xylia, Erik Olsson

**Affiliations:** 1 Stockholm Environment Institute, 115 23 Stockholm, Sweden; 2 Environmental and Energy Systems Studies, Lund University, 221 00 Lund, Sweden; 3 Department of Energy Technology, KTH Royal Institute of Technology, 100 44 Stockholm, Sweden

**Keywords:** Artificial Intelligence, Large Language Model, Study selection, Systematic maps, Systematic reviews

## Abstract

In this paper we show that OpenAI’s Large Language Model (LLM) GPT perform remarkably well when used for title and abstract eligibility screening of scientific articles and within a (systematic) literature review workflow. We evaluated GPT on screening data from a systematic review study on electric vehicle charging infrastructure demand with almost 12,000 records using the same eligibility criteria as human screeners. We tested 3 different versions of this model that were tasked to distinguishing between relevant and irrelevant content by responding with a relevance probability between 0 and 1. For the latest GPT-4 model (tested in November 2023) and probability cutoff 0.5 the recall rate is 100%, meaning no relevant papers were missed and using this mode for screening would have saved 50% of the time that would otherwise be spent on manual screening. Experimenting with a higher cut of threshold can save more time. With threshold chosen so that recall is still above 95% for GPT-4 (where up to 5% of relevant papers might be missed), the model could save 75% of the time spent on manual screening. If automation technologies can replicate manual screening by human experts with effectiveness, accuracy, and precision, the work and cost savings are significant. Furthermore, the value of a comprehensive list of relevant literature, rather quickly available at the start of a research project, is hard to understate. However, as this study only evaluated the performance on one systematic review and one prompt, we caution that more test and methodological development is needed, and outline the next steps to properly evaluate rigor and effectiveness of LLMs for eligibility screening.

**Supplementary Information:**

The online version contains supplementary material available at 10.1186/s13750-025-00360-x.

## Introduction

In 2021 a meta-review found 41 systematic reviews that used some form of automation across different review stages [1] growing to 52 in 2024 [2]. Indeed, there is a range of algorithms and machine learning-based tools readily available that can assist across the review stages [3] (For additional definitions and explanations of the concepts used here, see the SI, Glossary). In terms of conducting title and abstract screening, the most laborious first stage of systematic reviews, a lot of existing tools rely on pre-built or bespoke Machine Learning (ML) classifiers [4, 5]. However, these tools, sometimes referred to as discriminative artificial intelligence (AI) technologies, generally require human researchers providing large amount of sufficiently diverse data for training and active learning [1, 3, 5, 6]. With the recent proliferation of Large Language Models (LLMs)– a type of generative AI created by a deep learning neural network trained on a large written corpus (Berger-Tal et al. 2024)– models such as GPT and Gemini, have become publicly available and appear to markedly outperforms discriminative AI technology in applications and capabilities. Notably, the use of LLMs requires limited funds, technical expertise, and no additional training or fine tuning.

The potential usefulness of LLMs to process vast amount of scientific data and assist in systematic review process has until recently been nascent [2], but not been ignored by neither the systematic review community [7], nor environmental research scholars [8]. Spillas et al. [9] recently reported benefits from using GPT as a collaborative tool in systematic reviews, showing that AI can broaden the effectiveness of a systematic review’s search strategy. However, methods and tools for automation of systematic literature reviews have been mostly tested in the fields of medicine and computing, e.g., using ML for screening or Natural Language Processing (NLP) techniques for data extraction [1, 3] and in the last few years using LLMs [10–15]. Hence, there is fast growing literature evaluating how well automation works for screening in medical systematic reviews, and experiments with automated data extraction is again more frequent in medicine [16]. But we find almost no published studies using LLMs for abstract and screening in systematic reviews outside of medicine. The only exception we are aware of was a recent study by Nguyen-Trung and colleagues [17], which assessed the application of LLMs to assist with rapid reviews in a case study focused on land management and climate resilience. While the above literature show that LLM based methods can reach high performance in the field of medicine, it remains unclear if AI-assisted tools to automate screening in general, and LLMs in particular, perform as well in disciplines that have less standardized reporting formats and complex study designs, such as the fields of environmental and sustainability research.

In this paper, we explore the utility of readily available LLM for the initial study selection stage (title and abstract screening) of the systematic review process, and evaluate its performance compared to human screening.

## Methodology

We instructed the LLM to make a decision related to relevance of titles and abstracts based on a set of eligibility criteria. The records assessed by the LLM came from our recent systematic review of charging infrastructure demand that was completed without the support of AI (currently under peer review [18]. The review protocol is detailed in Macura et al. [19] and the review complied with guidelines and standards of Collaboration for Environmental Evidence [20]. It was conducted in the field of electrification technologies for vehicles, a relatively recent research area that is under fast development. Hence, this body of research offers a good case to test the utility of LLMs as the field where systematic review methodology has not been very established and review automation is not frequently tested and used.

To test LLM screening performance we used nearly 12,000 academic records that were originally manually screened. The manual screening of titles and abstracts was conducted by one reviewer. Before screening, and to assure consistency in screening decisions, a consistency checking exercise was performed on a sample of 100 records, which were independently screened by three reviewers. This exercise resulted in an interrater agreement of 88% in the first round, indicating high confidence in the screening process. Any doubts were discussed within the review team, and reviewers were advised to adopt an inclusive approach to screening.

For this validation study, human and the LLM screening decisions were then compared. We did not provide any additional training to the model, we did not fine-tune the model, or provided examples in the prompt. Three different versions of the GPT LLM (gpt-3.5-0311, gpt-3.5-0613, gpt-4-1106 - with release dates in 2023 incorporated in the model´s name) were instructed with the same prompt using the OpenAI API (see SI, Methodological Details, and SI, Box S2). Specifically, the LLMs were prompted to act as a reviewer, to apply the eligibility criteria (designed for our original review [19]) on a set of titles and abstracts, and to make a decision about their eligibility. The prompt specifically asked for the model response to include (1) a numerical assessment in the range 0 to 1 representing the level of probability that a given record is relevant (further referred to as ‘relevance probability’) with 0 being unlikely to be relevant and 1 being very likely, (2) a justification for the decision (see SI, Box S2). A researcher was then able to decide on the relevance probability score above which a record should be included (further referred to as the ‘probability cutoff’). Without any such decision or analysis, we refer to a default cutoff value relevance probability of 0.5 which can be interpreted as that record for the LLM is equally or more likely to be relevant, than not.

For the evaluation of performance, we test if a relevant record is correctly included. A Type I Error, a False Positive (FP), occurred if the LLM included irrelevant record. This error is less important as it only limits the potential of the work saved. A Type II Error, False Negative (FN), occurred if the LLM excluded a relevant record, which is the more important error (See also SI, Figure S1). Finally, we also calculated Specificity (S), Precision (P), Recall (R) and Work Saved over Sampling (WSS) (according to Cohen et al. [21]).

## Results of title and abstract screening using GPT API

Our findings show very promising performance (Fig. 1; Table 1). The LLMs show consistently high recall, defined as the proportion of true positives correctly identified by the model (see Table 1 and SI, Glossary), across versions near or at 100% for a relevance probability cutoff of 0.5. Moreover, it is clear that screening performance improves as new models are made available (Fig. 2). Specifically, the first version tested, gpt-3.5-0311 successfully screens out 1,100 of 11,984 titles and abstracts (9.5%) without any false negative errors (occurring when model excludes relevant studies) at a 0.3 probability cutoff. The first false negative error occurred at probability cutoff of 0.4 (Fig. 2b). Note that models in general generated increments of 0.1 in probability scores, and hence this is the resolution used when reporting results. For the second model version tested - gpt-3.5-0613, the performance increased to 2,300 titles and abstracts (18%) before the first false negative error (Fig. 2d) and at a cutoff probability of 0.7. Finally, gpt-4-1106 released late 2023 screens 6,700 (55%) (Fig. 2f) again with first errors at a cutoff probability of 0.7. The number of false positives errors (leading to inclusion of records that should be excluded) decrease from 10,700, to 9,600 and 5,300, again showing how the three models gradually improve screening outcomes. Notably, as the probability cutoff for which the first error occurs increased from 0.4 to 0.7 between the first and the third model, the confidence of the LLM to predict the correct screening increases over time.

**Table 1:** Performance of model gpt-4-1106 at different probability cutoffs. The performance is measured via specificity, sensitivity, precision, F-measure (Harmonic mean of P and R), and works saved over sampling metric (WWS) according to Cohen et al. [21]

Relevance probability scores	0	0.1	0.2	0.3	0.4	0.5	0.6	0.7	0.8	0.9	1
Specificity = TN / (TN + FP)	0.00	0.11	0.29	0.32	0.49	0.55	0.55	0.62	0.79	0.97	1.00
Recall (Sensitivity) R = TP/(TP + FN)	1.00	1.00	1.00	1.00	1.00	1.00	1.00	0.99	0.96	0.49	0.05
Precision (P) = TP/(TP + FP)	0.01	0.01	0.02	0.02	0.02	0.03	0.03	0.03	0.05	0.17	0.35
F-measure F = 2*P*R/(P + R)	0.02	0.03	0.03	0.03	0.04	0.05	0.05	0.06	0.10	0.25	0.09
WSS = (TN + FN)/N-(1.0-R)	0	0.11	0.28	0.32	0.48	0.55	0.55	0.61	0.75	0.48	0.04

Interestingly, the models are in general less conservative (have higher number of false negative errors) than humans performing title and abstract screening (Fig. 2a, c, e). That is, when model results are compared to the human full-text screening decisions (Fig. 2b, d, f) the model performance is much better (e.g., the first errors take place at cut off probability 0.1 (Fig. 2e) vs. 0.7 (Fig. 2f) for gpt-4-1106). Style and content of abstracts vary and might not include all important information, so when uncertainty arises, humans are instructed to be inclusive at title and abstract screening, retaining a conservative approach. The model consistently apply the same criteria and screens out papers at title and abstract stage that humans instead screen out at the subsequent full-text screening stage.

Overall, the potential work saved (sensu Cohen et al. (2006)), had GPT been used to assist in the underlying systematic review is thus substantial. At a recall rate of 100%, that is not allowing for any errors resulting from excluding papers that should have been included, the latest GPT 4 model tested in this study (GPT4 as of 6th Nov 2023) saves screening of more than 50% records from being screened by a human researcher. With a recall rate of > 95%, allowing for 5% false negative errors at a higher probability cutoff, the work saved could have been 75% (see SI for details). The work savings are thus higher than the older results for ML and NLP classifiers [21] and on par with more recent ML results with humans in the loop [5].

More experimentation and proper evaluation of the robustness of these results are clearly needed to further develop the method. Critically, developing a robust method for appropriately selecting the cutoff probability a priori is needed. A relevance probability scores of and above 0.5 as shown in Fig. 1 could be a good starting point, but judging from results shown in Fig. 2, a higher cutoff probability should be possible.

**Fig. 1:**
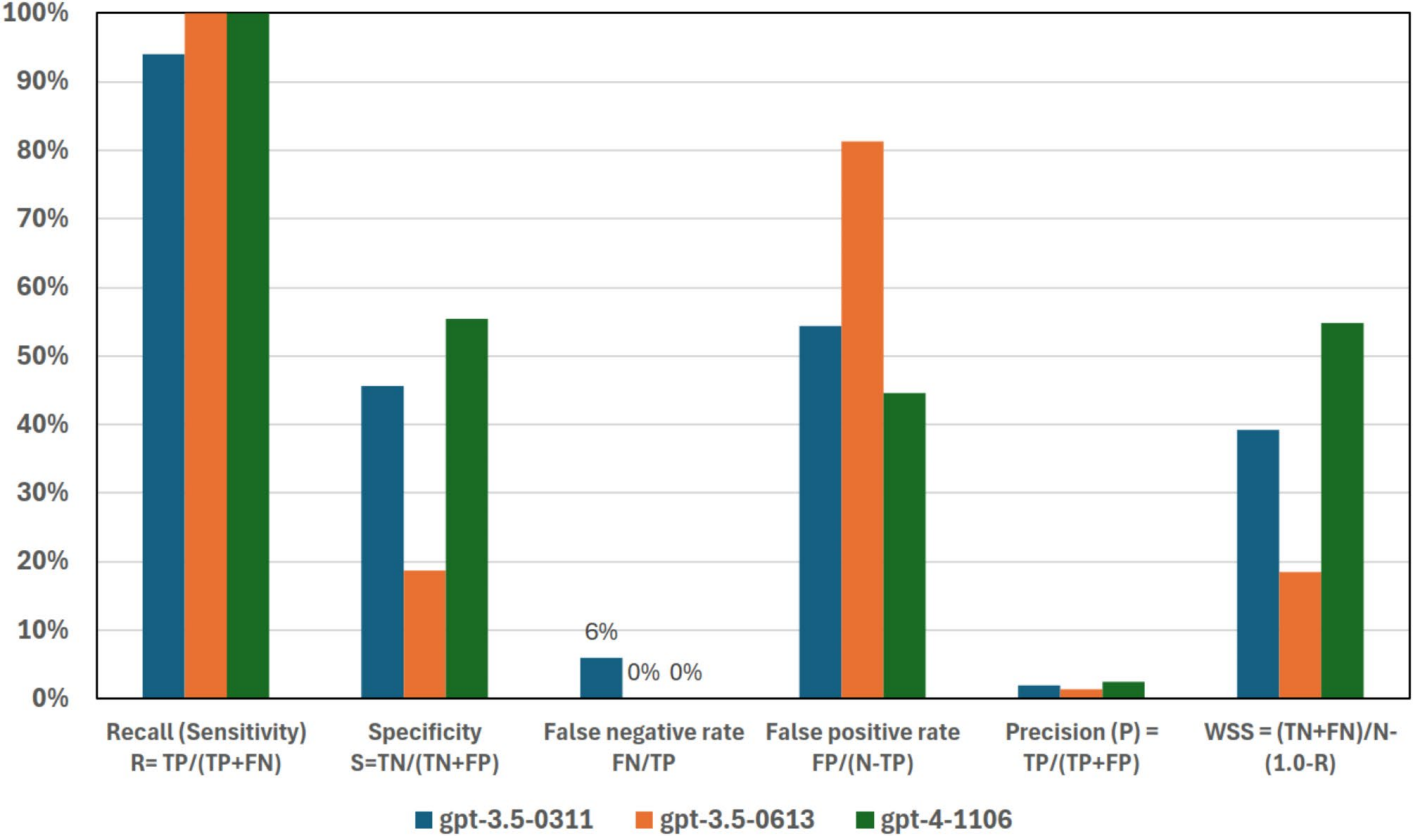
Performance across the three models at probability cutoff 0.5. False negative rate displays number to clarify that the value is zero for two of the models

**Fig. 2:**
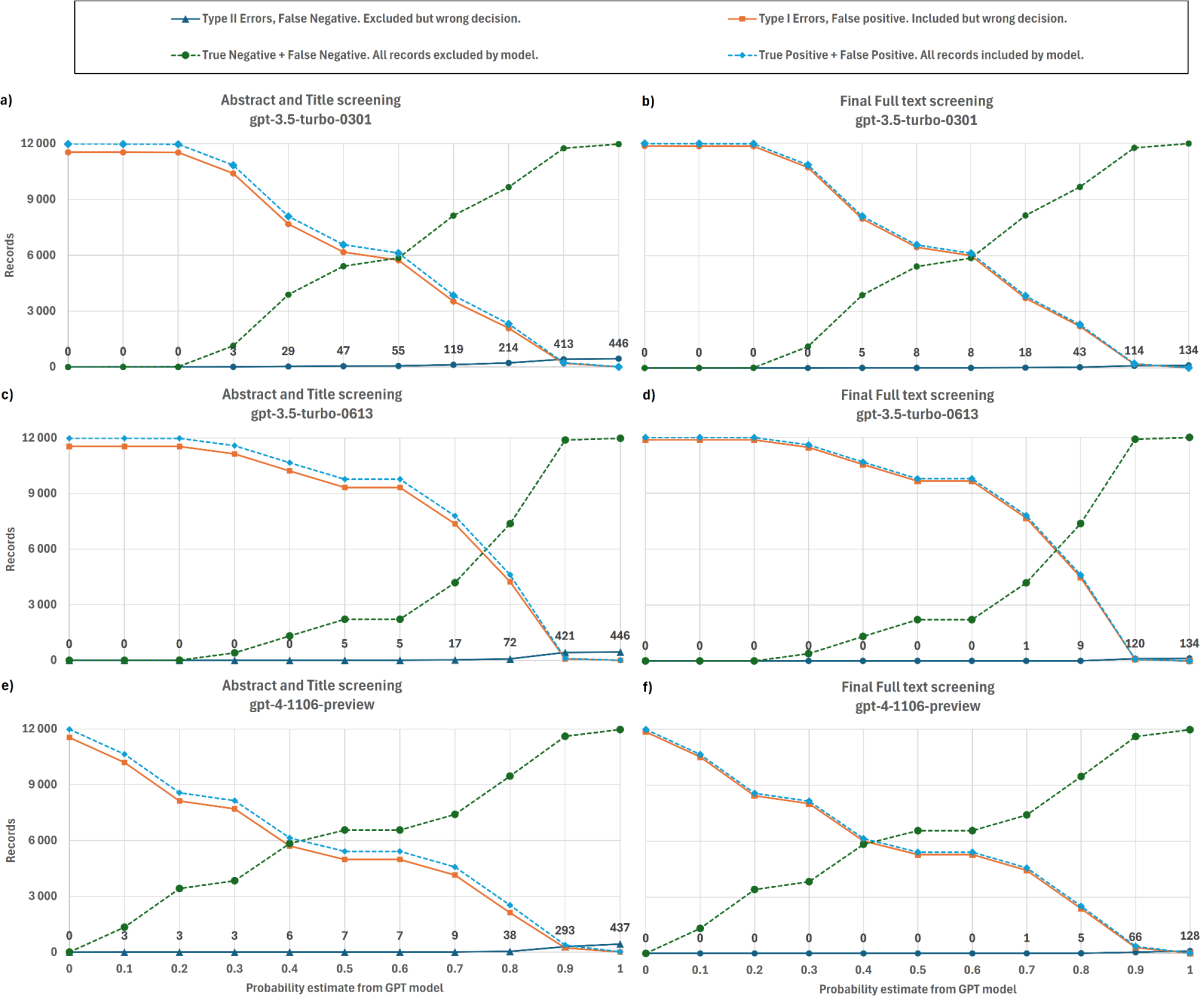
Performance in terms of number of records screened out by GPT, and the false positive errors (Type I) and false negative error (Type II) made as a function of the relevance probability. Columns in the panel show results for three versions of GPT model API used: Top row GPT3.5 as of 1st March 2023, second row, same model as of 13th June, and third row GPT4 as of 6th Nov 2023. Left hand side **a, c, e**, shows results benchmarked towards the results from title and abstract screening stage conducted by humans, and right-hand side **b, d, f**, compared to final set of included records in the review after full-text screening by humans. Numbers are shown above dark blue Type II errors lines for clarity

## Discussion

The good performance of LLMs for title and abstract screening shown in this paper illustrates the potential for wider application of LLMs as a tool for systematic reviews in general. Results we show here should, however, be interpreted with caution. For example, the constant adjustments and retraining of LLMs models by the developers, significantly lower the overall transparency and replicability potential for technology users. While the model provides explanations to each screening decision as instructed, the inner mechanism of a LLM is largely unknown and results are probabilistic. In addition, the rapid progress of LLMs with new versions frequently generated and old versions being depreciated, calls for more work and makes reproducing these early findings potentially a challenge. Reproducibility of the LLM application thus need to be ensured in order to increase usability of such models in scientific applications in general and in systematic evidence syntheses in particular [7, 22, 23]. We do note from the development and testing of the prompt used in this study that the model performs very similarly for consecutive test runs on a small batch of the same records (*N* = 100), but proper test of robustness would require iterative runs and more resources.

Furthermore, as we applied the models to just one specific field, and for one systematic review, the model performance could be connected to the style and reporting quality of titles and abstracts herein. Developing this approach further, tests should include evidence syntheses across multiple fields. Moreover, we show how performance depends on the relevance probability cutoff. Future assessments should include inquiry into what constitutes a robust probability cutoff based on a large range of systematic reviews, as the knowledge encoded in the LLM can be expected to vary across different fields. In general, a robust probability cutoff can potentially be dependent on the sample (e.g., how broad the search terms are), and the distribution of probabilities the LLM generates. In addition, more testing should evaluate how consistent LLM-based screening is compared to human screeners. As humans also make mistakes, this limits evaluation on performance in this initial test. Future validation studies should include several evaluation methods and metrics.

We could not test the LLMs performance on grey literature (which were included in the underlying review [18]), as a majority of them lacked abstracts, and hence could not be screened in the same way as academic studies (See, SI, Records with missing abstract). A more robust method needs to specify proper procedures to treat studies with varying formats, and the usefulness of LLMs on full-text screening should be explored, especially for reviews that include large amounts of grey literature. Since our experiments were conducted, efforts to extract information from full-text using LLMs are also accelerating (see e.g., https://www.sei.org/projects/developing-an-ai-powered-tool-for-data-extraction-from-texts/).

A notable result from the tests we presented here is that the LLM excludes records that humans would include at title and abstract stage but excluded at full-text screening, showing efficiency. More systematic experiments testing different prompts varying the instruction on how inclusive the model should be, need to be carried out. Moreover, since abstract and title screening decisions by the model agreed more with human screening decisions based on full-texts, the model could perform better if full-texts were immediately available to the model for screening. Nevertheless, processing full texts instead of bibliographic records would come at higher cost and less efficient workflow.

Although our early results indicate that the amount of work saved in terms of records that need to be screened is potentially high, the value of extending the option of automated screening methods to a broader range of disciplines with higher content and structure diversity is clear. This could enable faster and less costly systematic reviews in a range of subject areas in the field of environmental and sustainability studies. Any savings in laborious human screening not only enables elimination of human fatigue and bias [24], but would allow a larger number of primary research studies to be included in the screening, or, perhaps more useful– more time for human experts to focus on synthesis and interpretation of results.

## Conclusions

Using LLMs for screening of large amounts of title and abstract could have potentially saved more than 50% of worktime in our systematic review [18], without making a single false negative error. Accepting a recall rate of 95%, the work saved from using the latest GPT4 model from November 2023 is 75%. Given the fast improvement during 2023 and rapid development before GPT3 and 4, it can well be the case that LLM continues to improve, and the performance used for automated screening should thus only continue to increase. Since our approach performs well without additional training, this might imply that there is enough generic knowledge of the transport electrification field encoded in the pretrained LLM to effectively assist in research, but it is also a critical dependency, and since this technology is still in development, and transparency on training data sets is low, we argue for caution and more research. For now, humans in the loop are necessary [25].

Additional research on the robustness of these results is also motivated by known challenges of LLMs with fabricated knowledge, lack of transparency in both the training data and algorithm development as well as ethical concerns given biases related to gender and race [8, 25, 26]. But if the robustness of LLM results can be successfully tested against a range of historical systematic review studies, LLM can provide value in general for research processes, not only assisting in systematic reviews. Systematic review research projects can take several years to conduct, and LLM-assisted systematic reviews (screening tens of thousands of records in hours) thus has obvious benefits in general, and specifically to rapidly evolving research fields in environmental and sustainability studies.

## Electronic supplementary material

Below is the link to the electronic supplementary material.

Supplementary Material 1

## Data Availability

All data necessary to replicate results are available the Supplementary Information, also available in the Zenodo repository https://zenodo.org/uploads/15039995.
